# Low-Intensity Continuous Ultrasound Enhances the Therapeutic Efficacy of Curcumin-Encapsulated Exosomes Derived from Hypoxic Liver Cancer Cells via Homotropic Drug Delivery Systems

**DOI:** 10.3390/bioengineering11121184

**Published:** 2024-11-23

**Authors:** MinSeok Kim, YounJoong Kim, ChiYeon Hwang, MinHyeok Song, SuKang Kim, Kyung-Sik Yoon, InSug Kang, HyungHwan Baik, Yong-Jin Yoon

**Affiliations:** 1 Department of Medicine, Graduate School, Kyung Hee University, Seoul 02453, Republic of Korea; mskim9262@kaist.ac.kr (M.K.); sky9999@khu.ac.kr (K.-S.Y.); hhbaik@khu.ac.kr (H.B.); 2 Department of Mechanical Engineering, Korea Advanced Institute of Science and Technology (KAIST), Daejeon 34141, Republic of Korea; 3 Department of Structural Biology and Biophysics, University of Connecticut, Storrs, CT 06269, USA; younkim0306@gmail.com; 4 Department of Biomedical Science, Graduate School, Kyung Hee University, Seoul 02453, Republic of Korea; clehdrnfl@naver.com (C.H.); thdalsgur77@naver.com (M.S.); iskang@khu.ac.kr (I.K.); 5 Department of Biomedical Laboratory Science, Catholic Kwandong University, Gangneung 25601, Republic of Korea; skkim7@cku.ac.kr

**Keywords:** exosome, drug delivery system, low-intensity ultrasound, hypoxia, curcumin

## Abstract

Exosomes are extracellular nanovesicles secreted by cells that efficiently deliver therapeutic cargo for cancer treatment. However, because exosomes are present in low quantities and have limited target specificity, internal and external stress stimulation has been studied to increase exosome efficiency. Inspired by these studies, the uptake efficiency of cobalt chloride-induced hypoxic cancer cell-secreted exosomes was evaluated. Western blotting and RT-PCR data revealed increased exosome secretion and different protein compositions exhibited by hypoxic exosomes (H-Exos) compared to natural normoxic exosomes (N-Exos). Furthermore, these H-Exos were continuously stimulated using low-intensity ultrasound (LICUS) at an intensity of 360 mW/cm^2^ and a frequency of 3 MHz in vitro and 1 MHz in vivo. Hyperthermic and mechanical stress caused by ultrasound successfully improved exosome uptake via clathrin-mediated pathways, and confocal laser microscopy showed strong internal localization near the target cell nuclei. Finally, LICUS-equipped H-Exos were loaded with hydrophobic curcumin (H-Exo-Cur) and used to treat parent HepG2 liver cancer cells. The UV–Vis spectrophotometer displayed enhanced stability, solubility, and concentration of the encapsulated drug molecules. In MTT and FACS studies, approximately 40 times higher cell death was induced, and in animal studies, approximately 10 times higher tumor sizes were suppressed by LICUS-assisted H-Exo-Cur compared to the control. In this study, the delivery platform constructed demonstrated enormous potential for liver cancer therapy.

## 1. Introduction

Hepatocellular carcinoma (HCC) is the most common type of liver cancer and the fifth most common cancer worldwide [1]. Surgical resection and liver transplantation are recommended in the early stages of cancer. Nevertheless, only 5% to 15% of patients with HCC benefit from surgery due to poor prognosis. Treatment for advanced stages includes chemotherapy and immunotherapy. However, these options are associated with systemic toxicity, side effects, and drug inefficacy, especially when the drugs are delivered at incorrect locations. Therefore, diverse organic and inorganic nanomaterials have been investigated to reduce the off-target effects. Drug-loaded nanoparticles in the bloodstream are not easily discharged into the human body and are rapidly eliminated by phagocytosis, target specificity, and controlled-release therapy, revealing their enormous potential for drug delivery [2].

In recent years, naturally occurring cell-secreted nanoparticles called exosomes have received widespread attention as new-generation drug delivery platforms owing to their exceptional biocompatibility and ability to avoid mononuclear clearance mechanisms [3]. At 40–150 nm, exosomes can carry multiple biological components, including proteins, nucleic acids, lipids, and sugars, which can be transferred to recipient cells to mediate cell-to-cell communication [4]. Pioneering studies have explored the use of exosomes as drug delivery vesicles to deliver gene-/drug-encapsulated exosomes to target sites [5]. Despite this potential, the current exosome-based delivery systems are still in their infancy, with two major problems requiring further research: (A) the production of exosomes should meet sufficient abundance, and (B) naturally derived exosomes should further develop target specificity. Cancer cells in a hypoxic microenvironment increase the release of exosomes for survival [6]. Hypoxia also affects the properties of exosomes, which play a crucial role in cancer progression [7]. However, few studies have focused on the utilization of hypoxia-modified exosomes for drug delivery [8]. Moreover, exosomes are well known for their homotropic features, where vesicles can travel back to their mother cell lines [9]. This intrinsic ability offers an additional mechanism for extending cancer-targeting capability.

Exogenous stimuli have been implemented to control drug release precisely and utilize exosomes as site-specific NPs. Focusing ultrasound with microbubbles produces thermal, mechanical, and radiation forces that can stimulate drug particles near the exposed areas [10]. However, their relatively large size prohibits microbubbles from penetrating blood vessels and, therefore, into cancer cells [11]. To overcome this limitation, low-intensity ultrasound (LIUS) without cavitation or contrast agents has been used. LIUS-induced hyperthermia or mechanical stress promotes clathrin-mediated endocytosis, enhancing the penetration of drug molecules into tumor cells [12]. Based on this evidence, the role of low-intensity ultrasound, administered continuously (LICUS), in promoting exosome enrichment near the target sites was investigated.

Herein, this research demonstrates that we designed a delivery system for HCC therapy using exosomes derived from hypoxic HepG2 liver cancer cells combined with LICUS and curcumin. Curcumin, a component of *Curcuma longa* (turmeric plant), displays anti-inflammatory, antioxidant, and anti-cancer activities against multiple cancer cell lines [13]. Using exosomes increases the solubility, stability, and bioavailability of hydrophobic curcumin [14]. The anti-cancer effects of LICUS and curcumin-loaded exosomes were tested in homotropic HCC cancers. The combined drug delivery platform displayed unique advantages, including (i) utilization of naturally occurring exosomes with excellent biocompatibility and safety, (ii) sufficient hypoxia-induced exosome abundance, (iii) homotropic properties of exosomes derived from HCC cells enhanced target specificity and reduced off-target effects in in vitro and in vivo experiments, and (iv) LICUS-mediated non-invasive targeting specificity achieved promising therapeutic potential in clinical practice.

## 2. Materials and Methods

### 2.1. Materials

Dulbecco’s modified Eagle’s medium (DMEM), Roswell Park Memorial Institute medium (RPMI) medium, fetal bovine serum (FBS), and other cell culture products were purchased from Life Technologies (Grand Island, NY, USA). HepG2 (human liver cancer), SK-OV-3 (human ovarian cancer), AsPC-1 (human pancreatic tumor), RAW264.7 (mouse macrophage), and Chang (human normal liver) cell lines were purchased from the Korean Cell Line Bank (Seoul, the Republic of Korea). 3-(4,5-dimethylthiazol-2-yl)-2,5-diphenyl tetrazolium (MTT), propidium iodide (PI), TRI reagent, cobalt (II), chloride (CoCl_2_), curcumin (diferuloylmethane), Triton X-100, approximately 70–100 nm carboxylated polystyrene beads, and an Amicon^®^ Ultra-4 Centrifugal Filter Unit were obtained from Sigma-Aldrich (St. Louis, MO, USA). Antifade mounting medium containing DAPI solution was purchased from Vector Laboratories (Burlingame, CA, USA). Bovine serum albumin (BSA) was purchased from MP Biomedicals (Santa Ana, CA, USA). Total exosome isolation reagent (from cell culture media) was obtained from Thermo Fisher Scientific (Waltham, MA, USA). An Annexin V-fluorescein isothiocyanate (FITC) apoptosis detection kit was obtained from BD Biosciences (Oakville, ON, Canada). Antibodies against HIF-1α, caveolin-1, CD63, TSG101, cytochrome C, calnexin, clathrin, Rab27a, Rab7, β-actin, TIMP3, S100A4, and Bcl-2 were purchased from Santa Cruz Biotechnology (Dallas, TX, USA). Taq polymerase and reverse transcription-polymerase chain reaction (RT-PCR) reagents were purchased from Bioneer (Seoul, the Republic of Korea). Antibodies against Alix, Bax, Caspase-3, Bim, PARP, E-cadherin, N-cadherin, MMP-2, MMP-9, HRP-conjugated anti-mouse, HRP-conjugated anti-rabbit secondary antibodies, FITC-conjugated anti-mouse, and Cy3-conjugated anti-rabbit secondary antibodies were purchased from Cell Signaling Technology (Beverly, MA, USA). The enhanced chemiluminescence (ECL) system was acquired from Amersham (GE Healthcare, Piscataway, NJ, USA).

### 2.2. Exosome Characterization

#### 2.2.1. Exosome Isolation

Exosomes were isolated from supernatants of SK-OV-3, RAW264.7, and normoxic/hypoxic HepG2 cell lines. Briefly, cells were grown to 70% confluency in a 100 π culture dish before the medium was replaced with exosome-free 10% FBS medium. After 24 h, the medium used to separate exosomes was purified using syringe filtering to remove debris and apoptotic bodies. Exosomes were isolated using a Total Exosome Isolation Reagent kit (Thermo Fisher Scientific). The isolation was performed according to the manufacturer’s instructions. All samples were centrifuged using an Optima L-100 XP Ultracentrifuge (with an SW32Ti rotor) from Beckman Coulter (Brea, CA, USA), and the exosome pellets (kit isolates) were resuspended in PBS and stored at −80 °C before use.

#### 2.2.2. Exosome Size Exclusion Purification

Kit isolates (400 μL) were centrifuged at 1500× *g* and 10,000× *g* for 10 and 20 min, respectively, then overlaid onto a qEV size exclusion column (Izon Science Ltd., Cristchurch, New Zealand) and eluted with PBS. Fractions (400 μL) were collected, and particle and protein concentrations were measured using Tunable Resistive Pulse Sensing (TRPS) and the Bradford assay (Bio-Rad, Hercules, CA, USA), respectively. The high-particle and low-protein fractions of the kit isolates were pooled and concentrated on an Amicon^®^ Ultra-4 10 kDa nominal molecular weight centrifugal filter to a final volume of 200 μL.

#### 2.2.3. TRPS

The size distribution and concentration of the particles were analyzed with a TRPS (qNano, Izon Science Ltd.) using a NP100 nanopore at a 45-mm stretch. The concentration of particles was standardized using the multiple pressure calibration method with 80–100 nm carboxylated polystyrene beads at a concentration of 1.5 × 10^11^ particles/mL.

#### 2.2.4. Transmission Electron Microscope (TEM) Analysis

Briefly, 3 mL of exosome suspension was fixed in 100 μL of 2% paraformaldehyde, 2 μL of which was transferred onto each of two Formvar–carbon-coated electron microscopy grids. The grids were removed with stainless steel loops, and excess fluid was blotted gently on Whatman No. 1 filter paper. Grids were left to dry and stored in appropriate grid storage boxes. Grids were observed with a HT7800 transmission electron microscope (Hitachi, Japan) at 80 kV.

### 2.3. In Vitro Experiments

#### 2.3.1. Cell Culture and Hypoxia Exposure

HepG2, SK-OV-3, AsPC-1, RAW264.7, and Chang cells were cultured in DMEM and RPMI supplemented with 10% FBS, 100 U/mL penicillin, and 100 μg/mL streptomycin in a humidified 5% CO_2_ incubator at 37 °C. CoCl_2_, a commonly used hypoxia-mimetic agent, artificially induces hypoxia by blocking HIF-1α protein degradation. For hypoxia exposure, the HepG2 cells were serum-starved for 3 h and exposed to different concentrations (50, 100, 250, 500, and 750 μM) of CoCl_2_ at 37 °C for 24 h.

#### 2.3.2. MTT Assay

Cell viability was determined based on the conversion of MTT to MTT formazan by mitochondrial enzymes. Briefly, cells were seeded onto a 12-well plate at a density of 2 × 10^5^ cells/well in 1 mL of medium in triplicate, stabilized to grow, and then treated with various concentrations of CoCl_2_ (100, 250, 500, and 750 μM); curcumin; Pbs-Cur; N-Exo-Cur; and H-Exo-Cur, with or without ultrasound. After 24 h of incubation at 37 °C, 100 μL MTT solution (5 mg/mL stock) was added to the cells and incubated at 37 °C for 1 h. The medium was removed carefully, and 150 μL dimethyl sulfoxide was added to resolve the blue formazan in living cells. Finally, the absorbance was read at 540 nm with a SYNERGY HTX multi-mode reader (BioTek, Winooski, VT, USA).

#### 2.3.3. Western Blot Analysis

Cells (3 × 10^5^/well) in 3 mL of medium were incubated for 24 h at 37 °C in a 60 π culture dish. The cells were washed twice with ice-cold PBS, and the total cell lysates were prepared in a lysis buffer containing 50 mM Tris−HCl (pH 7.4), 150 mM NaCl, 1% Triton X-100, 50 mM NaF, 5 mM sodium pyrophosphate, 1 mM EDTA, 1 mM EGTA, 1 mM DTT, 0.1 mM PMSF, and 0.5% protease inhibitor cocktail. Whole-cell lysates were centrifuged (12,000× *g* for 10 min at 4 °C) to remove cellular debris. The protein concentration was determined using the Lowry method and a Bio-Rad DC protein assay kit. For Western blot analysis, cell lysates and purified exosomes containing equal amounts of protein (40 μg) were resolved using 8–15% SDS-polyacrylamide gel electrophoresis and then transferred onto nitrocellulose membranes. The blots were blocked with a solution containing 5% skim milk in Tris-buffered saline with 0.05% Tween 20 (TBST) for 1 h at room temperature and treated with primary antibodies in TBST overnight. Membranes were washed for 1 h with TBST and further probed with HRP-conjugated anti-mouse and anti-rabbit secondary antibodies in TBST for 1 h at room temperature. Finally, the immune complexes were visualized using an ECL detection system according to the manufacturer’s protocols.

#### 2.3.4. LICUS: Ultrasound Apparatus

For ultrasound treatment, the cells were seeded in a 60 π quartz culture dish and placed in the water above the transducer in a horizontal position. Ultrasound waves were produced using a Digisonic generator (Chungwoo Medical, Inc., Seoul, Republic of Korea). The frequencies of 1.0 MHz for in vivo and 3.0 MHz for in vitro experiments with 360 mW/cm^2^ intensity were treated for 15 min. The focal length was approximately 5 cm.

#### 2.3.5. Curcumin Encapsulation

Curcumin was encapsulated in SK-OV-3, RAW264.7, and normoxic/hypoxic HepG2 cell-secreted exosomes. To load a therapeutic drug with the exosomes (1 μg/μL), curcumin (25 μM in vitro and 30 μM in vivo) was gently mixed at a ratio of 1:1 *w*/*w*. To disrupt the exosome bilayer, sonication (Bandelin sonoplus HD 2070, Landsberger, Berlin, Germany) was performed at 20% amplitude, six cycles of 30 s on and 30 s off, with a three-minute cooling period between each cycle. Subsequently, the mixture was incubated at 37 °C for 1 h to allow the exosome membrane to recover. Moreover, the membrane of exosomes remained well preserved when curcumin was encapsulated in them via sonication (Figure S1). The curcumin encapsulation efficiency (EE) was calculated using the following formula:

$$\frac{Total\;amount\;of\;H\_Exo\_Cur-amount\;of\;curcumin\;in\;supernatnat}{Total\;amount\;of\;H\_Exo\_Cur}\times 100\%$$

The absorbance of curcumin inside the exosomes was determined at 450 nm using a SYNERGY HTX multi-mode reader (BioTek). Pbs-Cur and all Exo-Cur samples were mixed with an equal volume of PBS to achieve the final concentrations.

#### 2.3.6. Curcumin Release with or Without LICUS

N-Exo-Cur and H-Exo-Cur were placed in dialysis tubes to observe Cur release after encapsulation. Dialysis tubes contained 4 mL PBS at different pH values (pH 7.0 and 5.0) and 0.1% Triton X-100. All tubes were placed in a water bath at 37 °C. At each time interval, 200 μL of dialysate was collected and replaced with the same amount of fresh supplemented media. The concentration of released curcumin was detected by measuring the fluorescence with a SYNERGY HTX multi-mode reader (BioTek). In all the experiments, the cumulative release of the curcumin fraction was calculated based on a standard curve.

#### 2.3.7. Detection of Apoptosis Using Flow Cytometry

Apoptotic cell death was detected using flow cytometry and the annexin V-FITC/PI double labeling method. HepG2 cells were seeded at 2 × 10^5^ cells/mL in a 60 π culture dish and treated with Pbs-Cur, N-Exo-Cur, and H-Exo-Cur, with or without ultrasound. After incubation for 24 h, cells were harvested, washed twice with PBS, trypsinized, and collected using centrifugation. After resuspension in 100 μL 1 × binding buffer, the cells were incubated with 7.5 μg/mL annexin V-FITC and 7.5 μg/mL PI at room temperature in the dark for 1 h. The samples were analyzed using a Muse Annexin V & Dead Cell Kit and a Muse cell analyzer (Merck Millipore, Danvers, MA, USA).

#### 2.3.8. RT-PCR

Total RNA was extracted from cultured cells using the TRI reagent. cDNA was synthesized from 0.2 μg total RNA using M-MLV reverse transcriptase (Fermentas, Waltham, MA, USA). The specific primers for RT-PCR included the following: HIF-1α, forward 5′-CCCAATGTCGGAGTTTGGAAAA-3′ and reverse 5′-GCACCAAGCAGGTCATAGGT-3′; CD63, forward 5′-GCGGTGGAAGGA GGAATGAA-3′ and reverse 5′-AGCCCCCTGGATTATGGTCT-3′; Alix, forward 5′-CCCTG GATGTGATGGTGTCC-3′ and reverse 5′-CCTGTTGCTGTCCAAGTTGC-3′; and TSG101, forward 5′-CGGAGAGCCAGCTCAAGAAA-3′ and reverse 5′-CGGAGAGCCAGCTCAAGAAA-3′. Real-time PCR was performed using an ABI Prism 7500 Sequence Detection System (Applied Biosystems, Waltham, MA, USA) with SYBR-Green PCR Master Mix (Applied Biosystems). The PCR reaction was carried out for 40 thermal cycles. PCR products were visualized on a 1% agarose gel using ethidium bromide staining. GAPDH was used as a loading control.

#### 2.3.9. Migration Assay

HepG2 cells were seeded at a density of 5 × 10 cells/well in 24-well plates. After 24 h, a wound scratch was made using a 200-mL pipette tip on the cells treated with Pbs-Cur, N-Exo-Cur, and H-Exo-Cur, with or without ultrasound. The cells were maintained at 37 °C. All images were captured after 24 or 48 h to estimate the area occupied by the migratory cells. The migration capacity was calculated as Δarea/area (0 h) × 100%.

#### 2.3.10. Transwell Invasion Assay

For the Transwell invasion assay, the upper Transwell chamber was coated with growth factor-reduced Matrigel (Corning, Steuben, NY, USA). Then, 5 × 10^5^ HepG2 cells were diluted in 500 mL serum-free DMEM and treated with Pbs-Cur, N-Exo-Cur, and H-Exo-Cur, with or without ultrasound. DMEM containing 10% FBS was added to the lower chamber as a chemoattractant. After 24 h or 48 h, cells on the upper surface of the membrane were removed using a cotton swab, and invaded cells were fixed with 75% methanol for 30 min at room temperature, followed by 0.25% crystal violet staining (Sigma-Aldrich; Merck KGaA, St. Louis, MO, USA) for 1 h at room temperature.

#### 2.3.11. Confocal Microscopy

HepG2 cells were seeded at a density of 2 × 10^5^ cells/mL onto 12-well culture slides and allowed to adhere overnight. CD63-labeled or TSG101-labeled N-Exo-Cur and H-Exo-Cur, with or without ultrasound, and Pbs-Cur were cocultured with HepG2 cells for 12 h. After 12 h, cells were washed three times with PBS, fixed in 4% paraformaldehyde for 15 min, and blocked with a solution containing 1% BSA. The slides were then incubated with FITC-conjugated anti-mouse and Cy3-conjugated anti-rabbit secondary antibodies in PBS for 1 h at room temperature. The Pbs-Cur-associated mean fluorescence intensity was analyzed using a fluorescence spectrophotometer at an excitation wavelength of 488 nm and an emission wavelength of 530 nm. The slides were washed three times before staining with DAPI. Images were taken on a LSM-800 microscope (Carl Zeiss, Oberkochen, Germany) and analyzed using NIH ImageJ software (version 1.41). To study the internalization mechanism of the H-Exo-Cur-US, specific endocytic inhibitors, including chlorpromazine (CPZ; 30 μM), were used to pretreat the HepG2 cells for 1 h using the same confocal procedure.

### 2.4. In Vivo Experiments

#### 2.4.1. Animal Model (Xenograft)

Athymic nude mice (15, 5-week-old, female, 18–20 g body weight) were obtained from Koatech, Inc. (Gyeonggi-do, the Republic of Korea) and housed under controlled temperature (24 ± 1 °C), relative humidity (approximately 45–55%), and artificial 12-h light–dark cycle lighting, with water and standard food ad libitum. Zoletil 50 (tiletamine + zolazepam) and Rompun (xylazine) were purchased from VSP (Kyunggi-do, the Republic of Korea). Athymic nude mice were subcutaneously injected in the right flanks with 0.1 mL Matrigel and 0.1 mL HepG2 cells (1 × 10^7^ cells/mL). When their tumor reached 60–70 mm^3^, the tumor-bearing mice were randomly assigned to different groups and prepared for experimentation. Pbs-Cur or H-Exo-Cur was injected into the caudal vein. The first ultrasound was introduced at 0.5 h before H-Exo-Cur injection. A second ultrasonography was performed 10 h post-injection. This procedure was performed every 3 days. Tumor volume and weight, survival rate, and body weight were measured every 3 days. After 28 days of treatment, each tumor was fixed with 10% formalin and stored at 4 °C until analysis. All animal experiments were approved by the Committee of Animal Experiments of the College of Medicine, Kyung Hee University (KHSASP-22-380).

#### 2.4.2. Hematoxylin and Eosin (H&E) Staining

H&E solution, formic acid, and 3,3-diaminobenzidine-tetrahydrochloride hydrate (DAB) were purchased from Sigma-Aldrich. For histological observation, the tumors (Control, Pbs-Cur, and H-Exo-Cur-US samples) were resected and immediately fixed with 10% formalin, followed by dehydration with increasing concentrations of ethanol (70%, 96%, and 100%) and decalcification with 10% formic acid. The samples were embedded in paraffin, sectioned, and stained with H&E for morphological examination. The samples were treated with DAB and 0.5% H_2_O_2_ in PBS (pH 7.6). Imaging was performed using a fluorescence microscope (IX73; Olympus, Hachioji-shi, Tokyo, Japan) with a color CCD camera (DP80; Olympus).

### 2.5. Statistical Analysis

The experimental results are expressed as the mean ± standard error of the mean obtained from at least three independent experiments. Statistical significance was determined using one-way analysis of variance (ANOVA) and the R program suite (version 3.2.4; http://www.r-project.org, accessed on 1 October 2024). Statistical significance was set at *p* < 0.05. Individual *p*-values (* *p* < 0.05; ** *p* < 0.01) are indicated.

## 3. Results

### 3.1. Optimization of CoCl_2_ Dose to Induce Hypoxia in Hepg2 Cells

**Figure 1:**
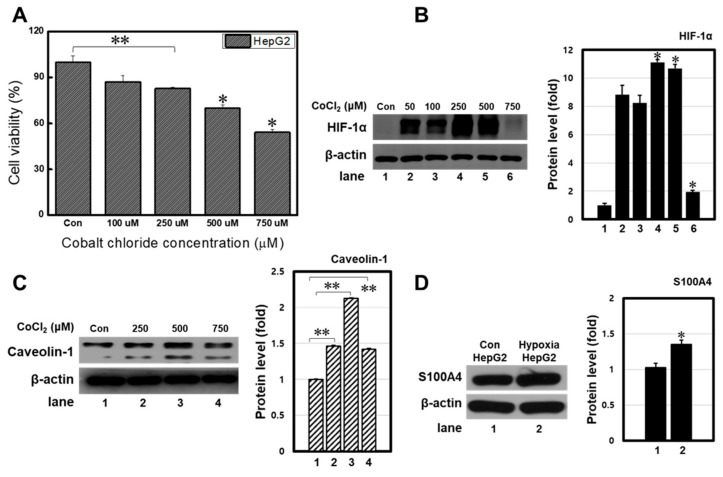
CoCl_2_-induced hypoxia on HepG2 cells. (**A**) Proliferation of HepG2 cells treated with different concentrations of CoCl_2_ confirmed by MTT assay, and 500 μM was selected in the follow-up study. Western blot analysis of hypoxia-related proteins (**B**) HIF-1α, (**C**) Cav-1, and (**D**) S100A4 on HepG2 cells treated with different concentrations of CoCl_2_. β-actin was used as the internal control. All proteins showed increased expressions when treated with 500 μM of CoCl_2_. ** *p* < 0.01 means comparison with the control group. * *p* < 0.05 means comparison with the control group. Values are the means ± SD of at least three independent experiments.

Cobalt chloride (CoCl_2_) artificially mimics hypoxia by blocking the degradation of HIF-1α protein. To investigate the effects of CoCl_2_ on cell viability, different concentrations of CoCl_2_ were treated against HepG2 cells in a dose-dependent manner. The cell viability reduced to 87.1 ± 4.1%, 82.8 ± 0.6%, 70.0 ± 2.2%, and 54.1 ± 1.8% after exposure to 100, 250, 500, and 750 μM of CoCl_2_, respectively (Figure 1A). To confirm CoCl_2_-induced hypoxia, the expression level of HIF-1α protein was observed using Western blotting. As hypoxia elevates the Caveolin-1 protein, which is linked to calcium-binding S100P, the expressions of Caveolin-1 and S100A4 were also investigated [15]. The HIF-1α and Caveolin-1 protein levels increased dose-dependently, indicating a hypoxic microenvironment in HepG2 cells (Figure 1B,C). Among different concentrations treated, 500 μM showed the highest HIF-1α and Caveolin-1 protein levels and also successfully elevated the expression of the S100A4 protein (Figure 1D). When treated with a concentration of 750 μM, most cells died, resulting in a weak band. Therefore, the concentration at 500 μM was selected for the follow-up study. In addition, after CoCl_2_ treatment, cells shrank, and apoptotic bodies were observed (Figure S2). These results indicated that CoCl_2_ induces hypoxia in HepG2 cells.

### 3.2. Characterization of Exosomes from Hypoxic (H-Exos) and Normoxic (N-Exos) HepG2 Cells

**Figure 2:**
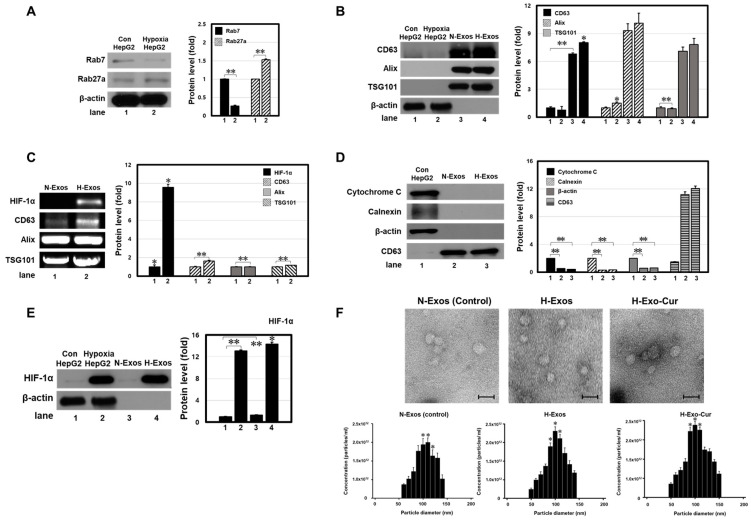
Isolation and characterization of exosomes. Western blot analysis of (**A**) exosome biogenesis-related markers (Rab7 and Rab27a); (**B**) common exosomal markers (CD63, Alix, and TSG101); (**D**) mitochondria and ER markers (cytochrome C and calnexin); and (**E**) hypoxia-related marker (HIF-1α) on control/hypoxic HepG2 cell lysate, N-Exos, and H-Exos. β-actin was used as the internal control. (**C**) The mRNA expression of hypoxia-related and common exosomal markers was measured by RT (reverse transcription)-PCR. In all cases, increased exosome secretion and different protein compositions/levels were found under a hypoxic environment. (**F**) TEM analysis images and TRPS analysis results of the exosome counts for each group: N-Exos, H-Exos, and H-Exo-Cur. TEM image scale bar: 200 nm. ** *p* < 0.01 means comparison with the control group. * *p* < 0.05 means comparison with the control group. Values are the means ± SD of at least three independent experiments.

Cancer cells generally produce more exosomes than non-cancerous cells due to nutrient deprivation and the need for intercellular information. Under hypoxic conditions and extreme nutrient and oxygen deficiencies, cancer cells increase exosome production for survival. To understand the molecular mechanism behind increased exosome production in HCC cancers undergoing hypoxic cellular stress, HepG2 cells were treated with 500 μM of CoCl_2_, and the expression levels of the proteins related to exosome biogenesis were analyzed using Western blots. Rab27a and Rab7, part of a large family of small GTPases, are key regulators of exosome secretion. Increased Rab27a and decreased Rab7 levels increased exosome production in ovarian cancer cells under hypoxia [16]. Consistent with the previous studies, upregulated Rab27a and downregulated Rab7 proteins in HepG2 cells were observed (Figure 2A).

Exosomes were collected from the H-Exos- and N-Exos-conditioned media of HepG2 cells. Common exosomal markers, such as CD63, Alix, and TSG101, were characterized at the transcriptional and translational levels to observe successful vesicle isolation. All proteins were elevated in both exosomes compared to those in the whole-cell lysate (Figure 2B,C). However, a noticeable elevation was observed in H-Exos compared to that in N-Exos. Considering that all samples were quantified using Western blot analysis, these data indicate that hypoxic conditions induced higher exosome production from HepG2 cells than that under normoxic conditions.

Proteins associated with the endoplasmic reticulum (ER) and mitochondria inside exosomes were analyzed. While HepG2 lysates showed cytochrome C and calnexin expression, no expression was observed in either exosome sample (Figure 2D). The lack of ER and mitochondrial proteins in the exosome preparations indicated that the isolated vesicles were free of cellular debris.

Exosome contents resemble many of their parent cell contents. Changes in the molecular context owing to hypoxia may be reflected in cell-secreted exosomes. To confirm the presence of exosomes under hypoxic conditions, a hypoxia-associated protein in H-Exos was analyzed. Previous results (Figure 1) confirmed an essential relationship between CoCl_2_-induced hypoxia and HIF-1α protein. Similarly, the HIF-1α protein was expressed in both H-Exos and hypoxic mother cells (Figure 2E). No expression was observed in normoxic HepG2 cells or N-Exos. As seen in the TEM and TRPS results of N-Exos (control exosomes), H-Exos, and H-Exo-Cur, the induction of hypoxia increased the number of exosomes compared to that in the control group (Figure 2F). Additionally, the average size of the collected exosomes ranged from 80 to 150 nm, and an increase in the number of exosomes was observed in the hypoxia-induced H-Exos and H-Exo-Cur groups.

These observations indicate hypoxia-induced alterations in liver cancer and how such stress affects cell-secreted exosomes. As the quantity of exosomes produced has always been an issue in exosome-based drug delivery, hypoxia might provide an alternative solution by increasing exosome production. In addition, the altered protein content inside H-Exos might promote enhanced uptake specificity towards target cells.

### 3.3. Exosomes Increase Solubility and Stability of Hydrophobic Curcumin

**Figure 3:**
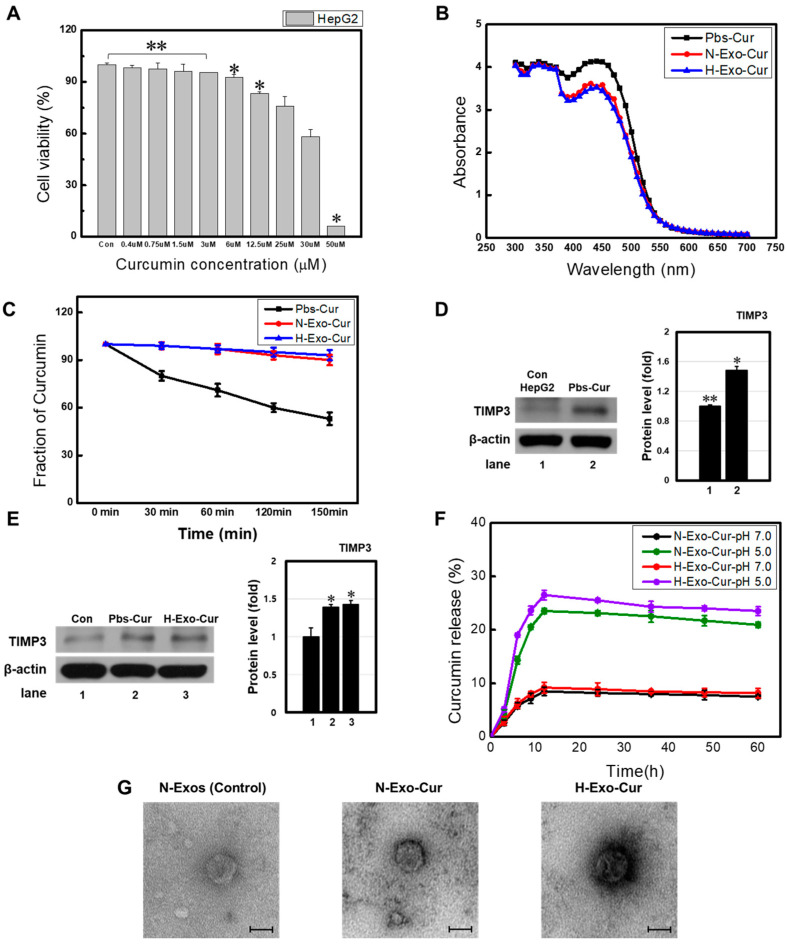
Preparation and characterization of Exo-Cur. (**A**) Proliferation of HepG2 cells treated with different concentrations of curcumin confirmed by MTT assay, and 25 μm was selected in the follow-up study. (**B**) The absorption and fluorescence spectra of either curcumin (Pbs-Cur) or N-Exo-Cur/H-Exo-Cur were measured using a UV–Vis spectrophotometer at 450 nm. Successful loading of curcumin inside the exosome was observed. (**C**) The concentration of curcumin was measured in a time-dependent manner. N-Exo-Cur/H-Exo-Cur both showed the highest stability. Western blot analysis of curcumin-related protein (TIMP3) on HepG2 cells treated with either (**D**) curcumin or (**E**) N-Exo-Cur/H-Exo-Cur. β-actin was used as the internal control. An increased concentration of curcumin was observed in the exosome samples. (**F**) The release profile of curcumin was from either N-Exo-Cur or H-Exo-Cur at different pH values (5.0 and 7.0). Increased curcumin secretion was observed from low pH. (**G**) TEM analysis images of the exosome for each group, N-Exos, N-Exos-Cur, and H-Exo-Cur. TEM image scale bar: 100 nm. ** *p* < 0.01 means comparison with the control group. * *p* < 0.05 means comparison with the control group. Values are the means ± SD of at least three independent experiments.

Curcumin is the hydrophobic component of *C. longa* and is used as a chemotherapeutic drug to treat colon, skin, and intestinal cancers. To determine the optimal concentration of curcumin, HepG2 cells were treated with curcumin in a dose-dependent manner (Figure 3A). Considering that too much cell death induced by curcumin might disturb the therapeutic results, 25 μM with approximately 20% cell death was selected. Next, 25 μM of curcumin was either encapsulated inside exosomes (H-Exo-Cur and N-Exo-Cur) or mixed in an equal volume of PBS (Pbs-Cur). The drug absorbance was measured using a UV–Vis spectrophotometer. The encapsulation efficiencies of the N-Exo-Cur and H-Exo-Cur samples were 52.33% and 57.25%, respectively. All three spectra showed no difference in the position or the shape of the absorbance band (Figure 3B). However, at 450 nm, the absorption spectra of the drug-encapsulated exosomes showed a much lower peak value than that of Pbs-Cur. Exosomal membranes might have disturbed the photon absorption of curcumin, indicating successful drug encapsulation.

Curcumin is more stable and soluble when encapsulated inside exosomes [14]. The stability of curcumin was measured, and a similar trend was observed (Figure 3C). During the same period, Pbs-Cur degraded quickly, whereas an almost-constant drug concentration was observed from drug-encapsulated exosomes (Pbs-Cur at 150 min, 53.2 ± 4.0% vs. H-Exo-Cur at 150 min, 93.2 ± 3.2%; Figure 3C). Next, HepG2 cells were treated with curcumin to determine the drug solubility. Chang et al. reported that curcumin inhibits HCC cells by downregulating miR-21 and upregulating TIMP3 expression [17]. Curcumin-treated HepG2 cells showed increased TIMP3 expression, indicating successful treatment (Figure 3D,E). This result indirectly supports the increased drug solubility when encapsulated inside exosomes. Increased solubility may have increased the drug concentration, enhancing TIMP3 expression.

Exosomes are incorporated into recipient cells through endocytosis and degraded within lysosomes. Lysosomes commonly exhibit pH 4–5 values, burst exosomal membranes, and release encapsulated drugs when internalized. To examine pH-dependent Cur release, H-Exo-Cur and N-Exo-Cur drug concentrations were observed periodically at different pH values (Figure 3F). At pH 7.0 (extracellular environment), H-Exo-Cur and N-Exo-Cur released less than 10% of the loaded curcumin within 60 h, and after 10 h, constant release profiles were observed. This suggests that curcumin was well protected inside the exosomes, and only a small amount of the drug leaked during blood circulation. In contrast, rapid and massive curcumin release was observed at pH 5.0 (lysosome), which was almost two to three times greater than that at pH 7.0. This indicates that drug-encapsulated exosomes enter cancer cells, where lysosomal degradation of the exosome membranes releases the drug, affecting cell viability. Figure 3G shows the TEM results of N-Exos (control exosome), N-Exo-Cur, and H-Exo-Cur; the size of the samples containing curcumin showed a slight increase or exhibited a black color surrounding the membrane, compared to that in the control group.

These results support the successful loading and enhanced therapeutic effects of curcumin-loaded exosomes compared to those of Pbs-Cur.

### 3.4. Parent HepG2 Cells Preferably Fused with Homologous Exosomes

**Figure 4:**
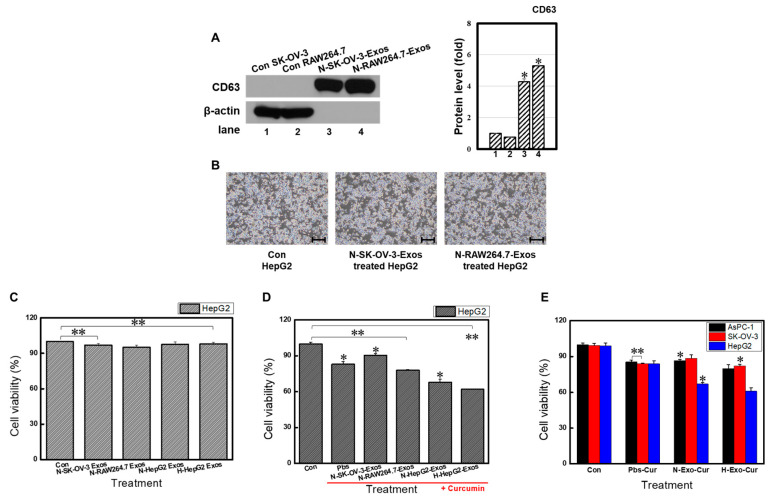
Homotropic activity of HepG2-derived exosomes. (**A**) Western blot analysis of CD63 on SK-OV-3 (ovarian cancer) and RAW264.7 (macrophage) cell lysate and derived exosomes. β-actin was used as the internal control. Successful exosome isolation was observed from both cells. Morphology (**B**) and viability (**C**) of HepG2 cells treated with exosomes from various origins. No differences were observed. The proliferation of (**D**) HepG2 cells or (**E**) multiple cancer cells treated with either Pbs-Cur or curcumin-loaded multiple exosomes was confirmed by MTT assay. H-Exo-Cur preferentially inhibited HepG2 cells. Microscopy image cale bar: 100 μm. ** *p* < 0.01 means comparison with the control group. * *p* < 0.05 means comparison with the control group. Values are the means ± SD of at least three independent experiments.

Homotropism, whereby secreted particles travel back to their parent cells, is a unique feature of exosomes [18]. To evaluate whether HepG2 cells prefer parent cell-derived vesicles, exosomes from the SK-OV-3 and RAW 264.7 cells were also isolated. All exosomes showed elevated levels of CD63, indicating successful isolation (Figure 4A). No exosomes exhibited cytotoxic effects on cancer cell viability or morphology (Figure 4B,C).

If exosomes induce no cytotoxic effect and the same amount of drug is deposited inside, the cancer cells with the highest uptake will experience the most cell death. These data indirectly indicate a homotropic targeting bias of particles secreted by liver cancer cells. Therefore, all exosomes were encapsulated with the same concentration of Cur, and the therapeutic effects of each Exo-Cur were compared (Figure 4D). As previously shown (Figure 3A), curcumin induced approximately 20% cell death (Pbs-Cur, 83.0 ± 2.3%; Figure 4D). Exo-Cur from normoxic liver cancer showed a higher cell death rate than those from ovarian cancer, suggesting special homotropic characteristics of parent cancer cells and derived exosomes (N-HepG2-Exos, 68.0 ± 2.4% vs. N-SK-OV-3-Exos, 90.4 ± 1.5%; Figure 4D). Macrophage-derived exosomes bear immunoprotective signals that decrease drug clearance before reaching the target, making them suitable for drug delivery [19]. Exo-Cur from immune cells was effective, as more cell death was induced than with Pbs-Cur (Pbs-Cur, 83.0 ± 2.3% vs. N-RAW264.7-Exos, 78.0 ± 0.5%; Figure 4D). Hypoxic exosomes showed the most significant inhibitory effect (N-HepG2-Exos, 68.0 ± 2.4% vs. H-HepG2-Exos, 62.1 ± 0.4%; Figure 4D). There are two possible explanations for these results. As mentioned above (Figure 2), exosomes from hypoxic cancer possessed increased amounts and concentrations of protein and altered protein composition. This may affect their ability to target cells. This result reinforces the enhanced exosome uptake in engineered parent cell environments.

To evaluate whether HepG2 exosomes preferred HepG2 cells and vice versa, the therapeutic effects of N-Exo-Cur and H-Exo-Cur on AsPC-1 and SK-OV-3 cells were compared. Exo-Cur from hypoxic liver cancer cells induced a more substantial apoptotic effect on parent cells than against other cancer cells (H-Exo-Cur-treated AsPC-1, 80.0 ± 3.3%, vs. H-Exo-Cur-treated SK-OV-3, 82.1 ± 1.6%, vs. H-Exo-Cur-treated HepG2, 61.5 ± 2.7%; Figure 4E). Minor differences were found between H-Exo-Cur and N-Exo-Cur against multiple cell lines, suggesting that the hypoxia-engineered targeting ability only functions when treating the parent cancer cells.

In all cases, homogeneous drug-encapsulating HepG2 exosomes showed the strongest suppressive effect against homogeneous HepG2 cell lines. Parent HepG2 cells and N-Exos or H-Exos are compatible and, therefore, offer the therapeutic potential for being a “Trojan horse”. HepG2 exosomes can travel back to their parent cancer cells to transport hydrophobic anti-cancer drugs efficiently.

### 3.5. LICUS Promotes Exosome Penetration Through Hyperthermia and Mechanical Stress

**Figure 5:**
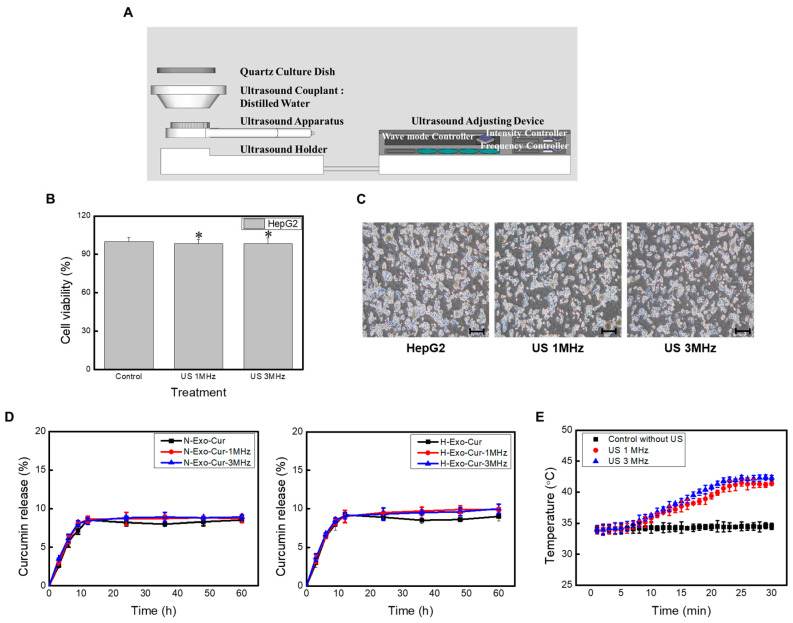
LICUS elevates exosomes uptake in vitro. (**A**) Schematic diagram of the ultrasound device. In vitro: 3 MHz with 360 mW/cm^2^ in 1 min duration. In vivo: 1 MHz with 360 mW/cm^2^ in 1 min duration. HepG2 Cell (**B**) viabilities and (**C**) morphology when treated with different ultrasound frequencies. (**D**) The release profile of either N-Exo-Cur (left) or H-Exo-Cur (right) with LICUS. Neither exosome showed any drug release with ultrasound stimulation. (**E**) Temperature measurement in cell culture Petri dishes in an incubator (black dotted line), with 1 MHz (red dotted line) or 3 MHz (blue dotted line) of ultrasound stimulation. Successfully, hyperthermia was induced from both frequencies. Microscopy image scale bar: 100 μm. ** *p* < 0.01 means comparison with the control group. * *p* < 0.05 means comparison with the control group. values are the means ± SD of at least three independent experiments.

LICUS is a non-invasive medical treatment that delivers low-intensity continuous waves. Low-intensity ultrasound treatment generally applies intensities ranging from 20 to 1000 mW/cm^2^ and frequencies ranging from 1 to 3 MHz [20,21]. In this experiment, an intensity of 360 mW/cm^2^ and frequencies of 1 and 3 MHz were used. A schematic of the LICUS apparatus is shown (Figure 5A). This frequency did not affect the cell viability or morphology (Figure 5B,C).

The interaction between ultrasound and contrast agents creates shockwaves and cavitation near the target area, leading to nanoparticle-encapsulated drug release and cell membrane permeabilization [22]. To determine whether LICUS assistance can boost drug release, the curcumin concentration was measured after ultrasound exposure. Briefly, H-Exo-Cur and N-Exo-Cur were placed in a quartz dish to minimize ultrasound disturbances and stimulated at frequencies of 1 or 3 MHz. As expected, without any contrast agents, the two frequencies had no additional effect on the drug release behavior (Figure 5D).

In this study, the interaction between LICUS and exosomes was irrelevant in the absence of CAs. However, LICUS offers mechanical and thermal stimuli when placed near the target lesion, increasing the drug uptake by cancer cells [20]. Therefore, the interaction between LICUS and the target cells was studied. Among the many biological effects of ultrasound, hyperthermia (mild temperatures around 43 °C) increases blood flow and vascular permeability, enhancing the delivery of chemotherapeutic drugs within the heated region [23]. To evaluate whether LICUS can produce mild heat, the local temperatures were measured after ultrasound treatment. Both 1 and 3 MHz successfully increased the temperature up to 40 °C, a defined temperature for hyperthermia (Figure 5E).

Nevertheless, 1 MHz and 3 MHz have different merits. A shorter wavelength (3 MHz) can generate more stimuli per given time, whereas 1 MHz with a longer wavelength can promote deeper penetration. In in vitro environments, where LICUS directly stimulates cells, 3 MHz with more stimulation cycles was more effective (the highest temperature was observed from 3 MHz). However, because deep penetration is required in vivo, 1 MHz was selected for subsequent in vivo experiments.

Next, to evaluate whether the interaction between LICUS and target cells promoted exosome uptake, confocal laser scanning microscopy was used to track the intracellular localization of exosomes (Figure S3). Cell nuclei were labeled with DAPI (blue), and exosomal membranes were labeled with the common exosome marker TSG101-FITC (green). Fluorescence microscopy confirmed that the exosomes were successfully fused within the parent cancer cells, as green fluorescence was well dispersed within the cytoplasm. As expected, the fluorescence of H-Exos was higher than that of N-Exos. Hypoxically engineered exosomes with homotropic features were preferentially taken up, and when camouflaged with hydrophobic drugs, they successfully attacked parent cancer cells.

Ultrasound treatment further enhanced exosome uptake. Notably, LICUS-stimulated N-Exos showed higher merged signals than those of H-Exos without stimulation. Exogenous stimuli increased the vesicle uptake more than innately induced endocytosis. Unsurprisingly, adding LICUS to H-Exos, which already showed excellent targeting efficiency, resulted in the highest cell localization. Compared to naturally secreted N-Exos, hypoxically modified H-Exos treated with LICUS showed almost twice the accumulation near the target cancer area.

The LICUS used in this experiment was not directly involved in the exosome and drug release behaviors. However, the multiple biological effects of ultrasound include targeting cancer cells and promoting vesicle uptake.

### 3.6. LICUS-Induced Exosome Uptake Is Mediated Primarily via the Clathrin-Mediated Pathway

**Figure 6:**
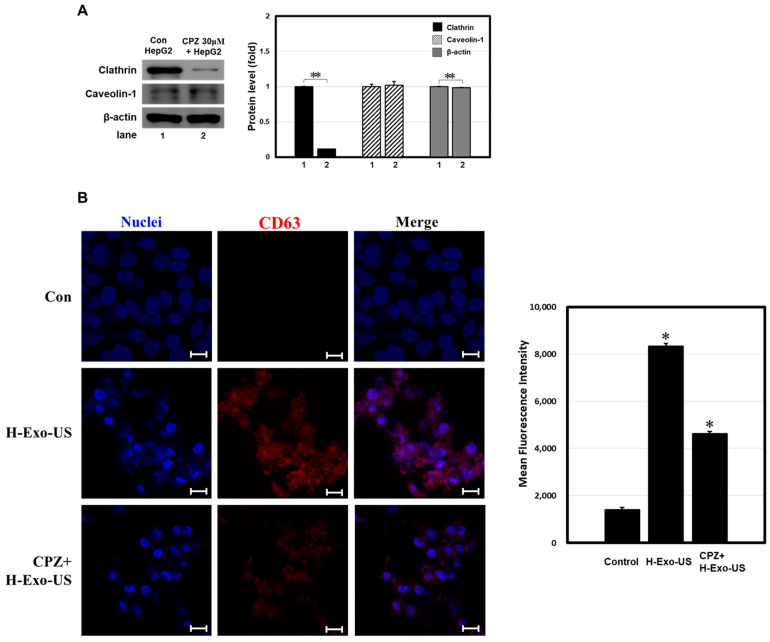
LICUS activates the clathrin-mediated pathway. (**A**) Western blot analysis of two important endocytosis pathways, clathrin and caveolin, in HepG2 cells with or without CPZ treatment. The clathrin-mediated pathway was successfully inhibited. (**B**) Uptake of dye-labeled H-Exos into HepG2 cells with or without CPZ treatment monitored by confocal laser scanning microscopy. Cell nuclei were labeled with DAPI (blue) signals, and exosomal membranes were labeled with common exosome marker CD63-Cy3 (red) signals. LICUS-triggered exosome uptake was mediated mainly by the clathrin pathway. Confocal microscopy image scale bar: 10 μm. ** *p* < 0.01 means comparison with the control group. * *p* < 0.05 means comparison with the control group. Values are the means ± SD of at least three independent experiments.

Clathrin forms round vesicles in the cytoplasm to sort cargo selectively near the cell membrane for intracellular trafficking. LICUS-triggered clathrin pathways effectively induced bisphosphonate uptake and anti-cancer activity in breast cancer cells [12,23]. To determine whether LICUS specifically acts on the clathrin pathway to induce exosome uptake, HepG2 cells were transiently treated with CPZ. Western blot analysis was used to characterize two important endocytosis pathways, clathrin and caveolin. As expected, CPZ-treated cancer cells showed reduced clathrin protein levels, whereas no difference was observed in the caveolin-1/caveolin-mediated pathway (Figure 6A).

Next, confocal laser scanning microscopy was used to validate the intracellular localization of the exosomes (Figure 6B). Cell nuclei were labeled with DAPI (blue), and exosomal membranes were labeled with the common exosome marker CD63-Cy3 (red). Similar to previous results (Figure S3), LICUS-triggered H-Exos were well dispersed within the cytoplasm, and purple (merged) fluorescence was easily observed. However, pre-incubation with CPZ after ultrasound stimulation reduced cellular uptake of H-Exos by up to 50%. When the LICUS-induced clathrin pathway was inhibited, cancer cells did not take up large numbers of exosomes. This indicated that clathrin-mediated endocytosis was dominant in LICUS-triggered cellular uptake, similar to a previously reported internalization mechanism of extracellular vesicles.

### 3.7. Cellular Uptake and Cytotoxicity of Homologous Hypoxic Exosome and LICUS

**Figure 7:**
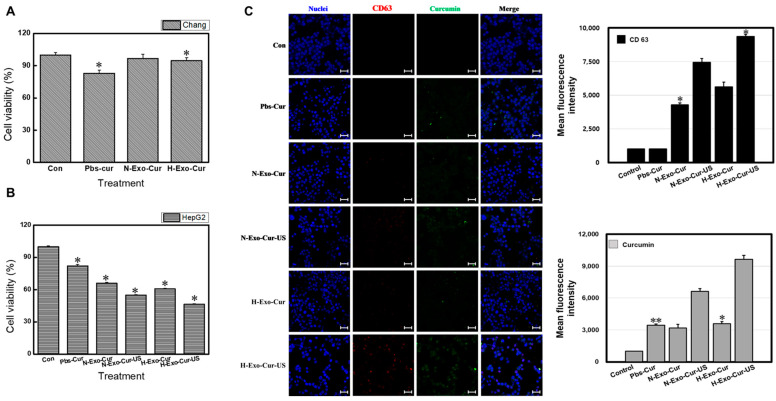
Therapeutic potentials of Exo-Cur with LICUS. The proliferation of (**A**) Chang normal liver cells or (**B**) HepG2 cells treated with either Pbs-Cur or curcumin-loaded multiple exosomes with or without ultrasound, confirmed by MTT assay. H-Exo-Cur and H-Exo-Cur-US showed the highest cytotoxic effect against the HepG2 cells, while no special effect was observed from the Chang cells. (**C**) Uptake of dye-labeled Exo-Cur into HepG2 cells by confocal laser scanning microscopy. Cell nuclei were labeled with DAPI (blue) signals, exosomal membranes were labeled with common exosome marker TSG101-FITC (green) signals, and pure curcumin was detected with 530 nm (green) signals after excitation at 488 nm. Curcumin-loaded exosomes were successfully located near cancer nuclei. Confocal microscopy image scale bar: 50 μm. ** *p* < 0.01 means comparison with the control group. * *p* < 0.05 means comparison with the control group. Values are the means ± SD of at least three independent experiments.

Off-target toxicity is a severe defect of conventional chemotherapy in clinical trials [24]. Similarly, a similar amount of apoptosis was induced in cells treated with Pbs-Cur, whether cancer (Figure 4D,E) or normal (Figure 7A): approximately 15.6%. This off-target effect is a significant barrier to the clinical efficacy of curcumin. Conversely, exosomes allow curcumin to reach specific targets. The designed Exo-Cur showed little or no effect on normal liver cells (Figure 7A), although the small amount of unencapsulated curcumin may have caused a negligible amount of cell death. Considerable cell death was induced in parent cancer cells (Figure 7B).

Exosome-based drug delivery systems exert anti-cancer effects based on intracellular accumulation. Exosome uptake near cancer cell nuclei, with or without ultrasound, has already been established (Figure S3 and Figure 6B). Therefore, the cellular uptake of curcumin-loaded exosomes was monitored using confocal microscopy (Figure 7C). Cell nuclei were labeled with DAPI (blue), exosomal membranes were labeled with the common exosome marker CD63-Cy3 (red), and pure curcumin was detected at 530 nm (green) after excitation at 488 nm. All Exo-Cur (N-Exo-Cur, H-Exo-Cur, N-Exo-Cur-US, and H-Exo-Cur-US) samples were preferentially taken up by HepG2 cells, and curcumin was successfully dissociated from the exosome and distributed well throughout the cytoplasm and nuclei of the cancer cells. Although the same amount of drug was loaded into all Exo-Cur samples, the cells with the highest exosome uptake showed the highest amount of curcumin (curcumin dissociation) and green fluorescence.

A relatively high fluorescence intensity was observed for Pbs-Cur. The Pbs-Cur used in this study was directly administered to the cancer cells. In clinical studies, after oral administration, curcumin is digested in the stomach, passes through the intestinal walls and into the liver for detoxification, and finally, enters the bloodstream. During this process, only a small amount of curcumin (compared to the initial dose) is delivered to the target cancer. Exosomes increase the stability and solubility of hydrophobic curcumin [14]. Most importantly, curcumin encapsulated inside exosomes maintained a similar concentration to the initial concentration; therefore, a higher amount of curcumin was dispersed within the cell nuclei, as shown using confocal microscopy. These results demonstrate the excellent therapeutic potential of the designed nanosystem against liver cancer cells.

In this study, H-Exos showed more precise targeting of HepG2 cells than N-Exos. With LICUS, H-Exos were preferentially located near the cell nuclei, and H-Exo-Cur induced more apoptosis than N-Exo-Cur. Therefore, in the follow-up study, N-Exo-Cur was not investigated with LICUS. However, N-Exo-Cur was investigated for comparison to H-Exo-Cur.

### 3.8. LICUS Accelerates Anti-Cancer Activity of H-Exo-Cur In Vitro

**Figure 8:**
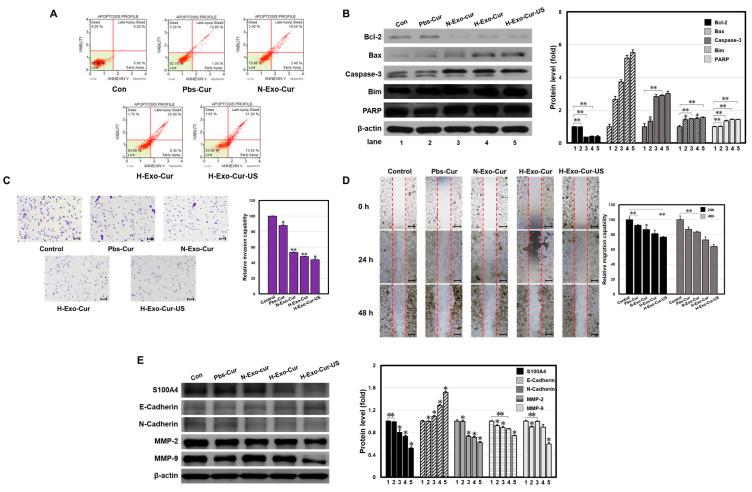
LICUS assisted H-Exo-Cur on cancer drug delivery. (**A**) Apoptotic cell death detected by annexin V-FITC/PI double staining. Drug-induced early/late apoptotic and necrotic cell death were detected. (**B**) Western blot analysis of apoptotic proteins (Bcl-2, Bax, Caspase-3, Bim, and PARP) of HepG2 cells after drug treatment. β-actin was used as the internal control. In all cases, similar apoptotic trends were observed. (**C**) Transwell assays of drug-treated HepG2 cells: cells invaded through Matrigel were stained with crystal violet and observed with a microscope. (**D**) Wound-healing assays of drug-treated HepG2 cells were incubated for 24 h and 48 h. The dotted red line separates cell layers (left, right) and the space occupied by migrated cancer cells (middle). (**E**) Western blot analysis of metastasis/migration-associated genes (S100A4, E-Cadherin, N-Cadherin, MMP-2, and MMP-9) of HepG2 cells after drug treatment. β-actin was used as the internal control. Overall, suppressed invasion/migration was observed. Microscopy image scale bar: 10 μm. ** *p* < 0.01 means comparison with the control group. * *p* < 0.05 means comparison with the control group. Values are the means ± SD of at least three independent experiments.

To examine how Exo-Cur induces cancer cell death, annexin V-FITC/PI double staining was performed (Figure 8A). The rate of early apoptosis in cells treated with H-Exo-Cur was approximately eight times higher than that in cells treated with Pbs-Cur. When ultrasound was used, the percentage increased from 8.35% to 13.55%. Likewise, the late apoptotic cell rate showed approximately two times the increase of Pbs-Cur-treated cells. With ultrasound, the percentage grew from 25.9% to 31.3%. Compared to early or late apoptosis, H-Exo-Cur, with or without ultrasound, had a small or negligible effect on the necrotic cell rate. Next, we determined the expression levels of proteins that play vital roles in apoptosis (Figure 8B). Ultrasound treatment with H-Exo-Cur upregulated Bax, Caspase-3, Bim, and PARP expression and downregulated Bcl-2 expression.

Metastasis, in which cancer cells travel away from their original tumor and form a new tumor in other organs or tissues, is a complex challenge faced by all cancer-targeting drugs. The anti-cancer effects on cancer cell invasion and migration were also measured. To measure cell invasion, a Transwell assay was performed (Figure 8C). The group treated with H-Exo-Cur and ultrasound showed a minimum number of invasive cells, approximately 43.7% less than that with Pbs-Cur. To evaluate cell proliferation, wound healing and scratch assays were performed (Figure 8D). Setting the dotted red line as the base, the number of samples that crossed the line was fewer than that in the control. Moreover, the proliferation rate of all samples was suppressed by approximately 1.5 times more at 48 h compared to that at 24 h. As expected, H-Exo-Cur with ultrasound showed the most suppressive effect; the migration of HepG2 cells was reduced by approximately 15.9% and 22.8% compared to that with Pbs-Cur at 24 h and 48 h.

Cancer cell migration and invasion are often linked to epithelial–mesenchymal transition (EMT). EMT markers were determined based on the expression of migration-associated proteins (Figure 8E). Without treatment, the liver cancer cells showed strong expression of metastatic (S100A4) and MMP (MMP-2 and MMP-9) markers. The hallmarks of EMT suppression are the upregulation of E-cadherin and downregulation of N-cadherin [25]. Pbs-Cur-treated samples showed no noticeable effects on these proteins. After treatment with H-Exo-Cur and ultrasound, the expression of metastasis and MMP markers was significantly suppressed, that of the epithelial marker (E-Cadherin) was upregulated, and that of the mesenchymal marker (N-Cadherin) was downregulated.

In all cases, LICUS-triggered H-Exo-Cur showed anti-cancer effects in vitro. The preferred uptake via homotropism and increased uptake and enhanced internalization by hypoxic engineering with hyperthermic and mechanical stress via LICUS successfully induced cancer cell death and suppressed cancer cell invasion and migration.

### 3.9. LICUS Combined H-Exo-Cur Effectively Improves Anti-Cancer Activity In Vivo

The ability of hypoxia to increase exosome production and selectively target homotropic cancers using LICUS offers therapeutic opportunities for liver cancer treatment. To test this hypothesis in vivo, the cancer-targeting ability of H-Exo-Cur with LICUS was investigated using athymic nude mice bearing HepG2 cells.

**Figure 9:**
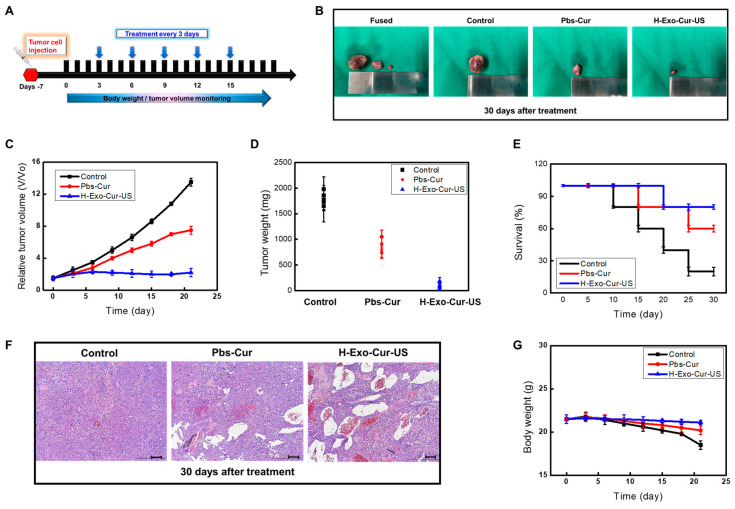
LICUS-assisted H-Exo-Cur inhibits liver cancer in mice. (**A**) Diagram representing the in vivo experimental procedure. (**B**) HepG2 cancer tissues were obtained from the indicated treatment groups. Control: 2 ± 0.2 cm, Pbs-Cur: 1 ± 0.3 cm, and HEC-US: 0.5 ± 0.1 cm. (**C**) Volume and (**D**) weight of HepG2 cancers in nude mice during the period of drug dosage. Cancer volume/weight was evaluated every 3 days for 30 consecutive days. Reduced cancer tissue was observed visually or numerically. (**E**) Survival curves of the in vivo treatment study. Longer survival times were observed compared to the control. (**F**) H&E staining of HepG2 tissue. H&E staining revealed apparent tissue damage caused by different treatments. (**G**) Effects of different treatments on in vivo body weight. No major changes were caused in response to the following curcumin treatment.

Cancer development was monitored, and treatment protocols were initiated as previously described (Figure 9A). Briefly, exosomes were injected after tumor implantation, with a curcumin concentration of 30 μM every 3 days for 30 days. To maximize ultrasound exposure, LICUS was applied twice: 0.5 h before H-Exo-Cur injection and at 10 h post-H-Exo-Cur administration. On the last day of the treatment, both the Pbs-Cur and H-Exo-Cur groups inhibited cancer growth compared to that of the control group (Figure 9B–D). The H-Exo-Cur-US treatment showed the most regression in tumor growth and an extended survival time (Figure 9E), improving hydrophobic curcumin treatment. Exo-Cur was concentrated near the target area, whereas Pbs-Cur was exposed to many different body parts, causing various side effects. In summary, H-Exo-Cur combined with two LICUS treatments in specific areas inhibited tumor growth to a greater extent and improved the therapeutic effect of the original curcumin treatment.

H&E staining was performed on tumors collected from mice to evaluate their in vivo anti-cancer effects (Figure 9F). The results further confirmed the superior anti-cancer efficacy of H-Exo-Cur with LICUS, and H&E staining revealed the apparent tissue damage caused by Exo-Cur injection.

The safety profiles of the designed nanotherapy were analyzed based on weight changes and the survival period of the mice. The body weight of the control group decreased during this period, whereas no significant changes were observed in response to the curcumin treatment (Figure 9G). Taken together, these data provide a relatively safe evaluation and indicate a high therapeutic efficacy of the designed nanoplatforms.

## 4. Discussion

In this study, exosomes were reprogrammed by inducing hypoxia in parent cancer cells and continuous stimulation using low-intensity ultrasound. Such modifications and stimulation substantially improved exosome targeting and uptake efficiency. H-Exos were successfully fused with hydrophobic curcumin and effectively delivered into homotropic liver cancer cells using LICUS. Thus, the nanoplatform designed in this study provides a valuable strategy for cancer-specific targeted therapies.

Hypoxia occurs frequently in cancer cells. Uncontrolled cell proliferation and abnormal tumor vessels reduce oxygen and nutrient transport, resulting in a hypoxic environment. The biological contents and effects of exosomes from hypoxic HCC cells have been investigated. miR-1273f is upregulated in hypoxic HCC exosomes, enhancing the malignant phenotype of normoxic HCC cells [6]. Upregulated miR-155 and miR23a in hypoxic HCC exosomes are taken up by HUVECS, promoting angiogenesis [26,27].

In this study, we observed increased exosome secretion and altered protein composition. Hypoxic and normoxic HepG2 cells showed different levels of Rab7 and Rab27a. Compared to those in naturally occurring N-Exos, hypoxically engineered H-Exos showed elevated levels of common exosomal markers (CD63, Alix, and TSG101). HIF-1α contained in hypoxic HepG2 cells was also discovered inside secreted exosomes. H-Exos accumulated near the cell nuclei/cytoplasm, and with LICUS, H-Exos showed almost twice the accumulation near the target cancer area compared to that with N-Exos. When loaded with curcumin, H-Exo-Cur exhibited stronger anti-cancer effects, with more curcumin near the cell nuclei/cytoplasm, more apoptotic cells, and significant suppression of cancer invasion or migration.

While most of this research has focused on the application and phenomena of these altered exosomes near the target area, the underlying structural alterations or mechanisms behind exosomes changed under hypoxic conditions must be further investigated. Gong. et al. engineered natural MGC803 human gastric cancer exosomes by inducing an acidic (low pH) microenvironment in donor cells. Lipidomic analyses have revealed different lipid structures (especially a higher glycerolipid composition) in these low-pH exosomes, impacting homologous interactions with target cells [28]. Therefore, more in-depth research on lipid structural analysis or membrane proteins involved in hypoxic exosomes could help explain the mechanism underlying exosome uptake in specific organs.

The mechanism of uptake between the parent and homologous cancer cells remains unclear. Exosomes may be particularly effective in targeting parent cells, as their protein content and composition are similar to those of the parent cells. Cancer exosomes fuse with cancer cells through lipid mixing, unique protein/receptor composition, or exosomal integrins [28,29]. However, not all exosomes have homotropic properties. Some researchers have reported a similar accumulation of exosomes in their parent cells and cells of other origins; therefore, the ability of homogenous exosomes to specifically target their parent cell line may be limited [30]. The biological properties of exosomes from various source cells are still being unraveled and need to be standardized.

In this study, homogenous HepG2 exosomes were preferentially taken up by parent HepG2 cells. Both naturally occurring N-Exos and hypoxically engineered H-Exos are located near the nuclei/cytoplasm of cancer cells. When loaded with curcumin, N-Exo-Cur and H-Exo-Cur showed the greatest suppressive effects against parent HepG2 cells compared to those against other cancer or normal cell lines. HepG2 cells preferentially fused with HepG2 exosomes. Compared to (curcumin-encapsulated) ovarian cancer or macrophage immune cell-secreted exosomes, N-Exo-Cur or H-Exo-Cur induced a more substantial apoptotic effect against parent liver cancer cells. In vivo studies showed reduced cancer volume/weight, and H&E staining revealed cancer tissue damage caused by Exo-Cur injection. No noticeable changes in body weight or a longer survival period were observed in Exo-Cur-treated mice, indicating the safety of the designed nanoplatforms.

In vivo and in clinical studies, the biodistribution, metabolism, and clearance of exosomes remain major issues in exosome-based drug delivery. Despite its therapeutic efficacy, the potential adverse effects require further investigation. Wang et al. performed H&E staining of the heart, liver, spleen, and kidney after treating blood serum-derived B-Exos (doxorubicin-loaded) against the GL261 glioblastoma cell line [31]. All major organs of the Exo-Dox-treated mice were similar to those of the untreated control groups, and no significant pathological changes were observed. Cheng et al. detected the biodistribution of exosomes in HT1080 fibrosarcoma and HeLa cervical cancer cells using IVIS imaging systems [32]. HT1080 exosomes were preferentially localized to the tumor tissue, whereas HeLa exosomes were abundant near other organs. Further studies should investigate the biodistribution of HepG2 exosomes to determine whether H-Exos/N-Exos can sufficiently colonize the parent cancer site and other organs.

## 5. Conclusions

These findings indicate the therapeutic effects of exosomes derived from hypoxic liver cancer cells loaded with hydrophobic curcumin and that low-intensity ultrasound can further enhance exosome uptake. A different method that utilizes ultrasound to promote vesicle endocytosis has also been investigated. The clathrin pathway activated by low-intensity ultrasound was not related to microbubble–cell interactions. Mild hyperthermia (between 42 °C and 43 °C) and mechanical stimulation via LICUS were sufficient to promote exosome penetration. This is the first study to report the relationship between low-intensity ultrasound and hypoxic exosome-based drug delivery systems. The designed platform provides a valuable strategy for treating other cancer-related diseases.

## Data Availability

Datasets are available upon request.

## Supplementary Materials

The following supporting information can be downloaded at https://www.mdpi.com/article/10.3390/bioengineering11121184/s1: Figure S1: TEM analysis image of the exosome counts for each group; Figure S2: HepG2 cell morphology treated with or without 500 μM of CoCl_2_; Figure S3: Uptake of dye-labeled exosomes into HepG2 cells monitored by confocal laser scanning microscopy.
