# Supplementary material for: Low-Intensity Continuous Ultrasound Enhances the Therapeutic Efficacy of Curcumin-Encapsulated Exosomes Derived from Hypoxic Liver Cancer Cells via Homotropic Drug Delivery Systems
Source: Bioengineering (Basel). 2024 Nov 23;11(12):1184. doi: 10.3390/bioengineering11121184 (PMC11673775; doi:10.3390/bioengineering11121184)
Supplement: Supplementary file 1 [file bioengineering-11-01184-s001.zip › bioengineering-3273901-supplementary.pdf]

Fig S1

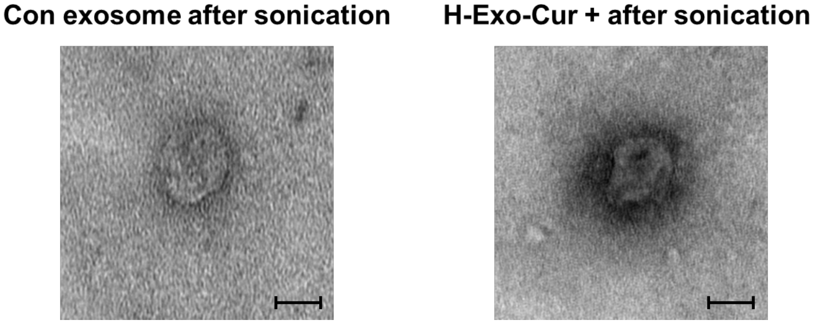

TEM analysis images of the exosome counts for each group, N-Exos after sonication and H-Exo-Cur after sonication. TEM image scale bar : 100 nm.

Fig S2

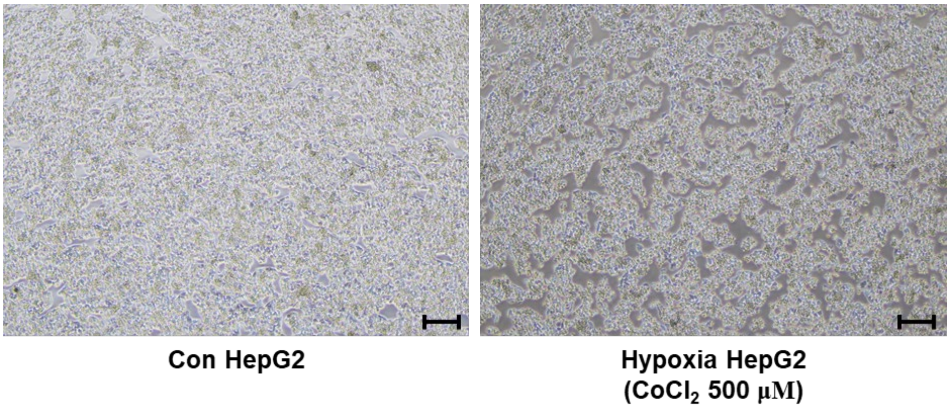

HepG2 cell morphology treated with or without 500 μM of $CoCl_2$. Shrinked morphologies and apoptotic bodies from hypoxic HepG2 cells were observed. Microscopy image scale bar : 100 μm

Fig S3

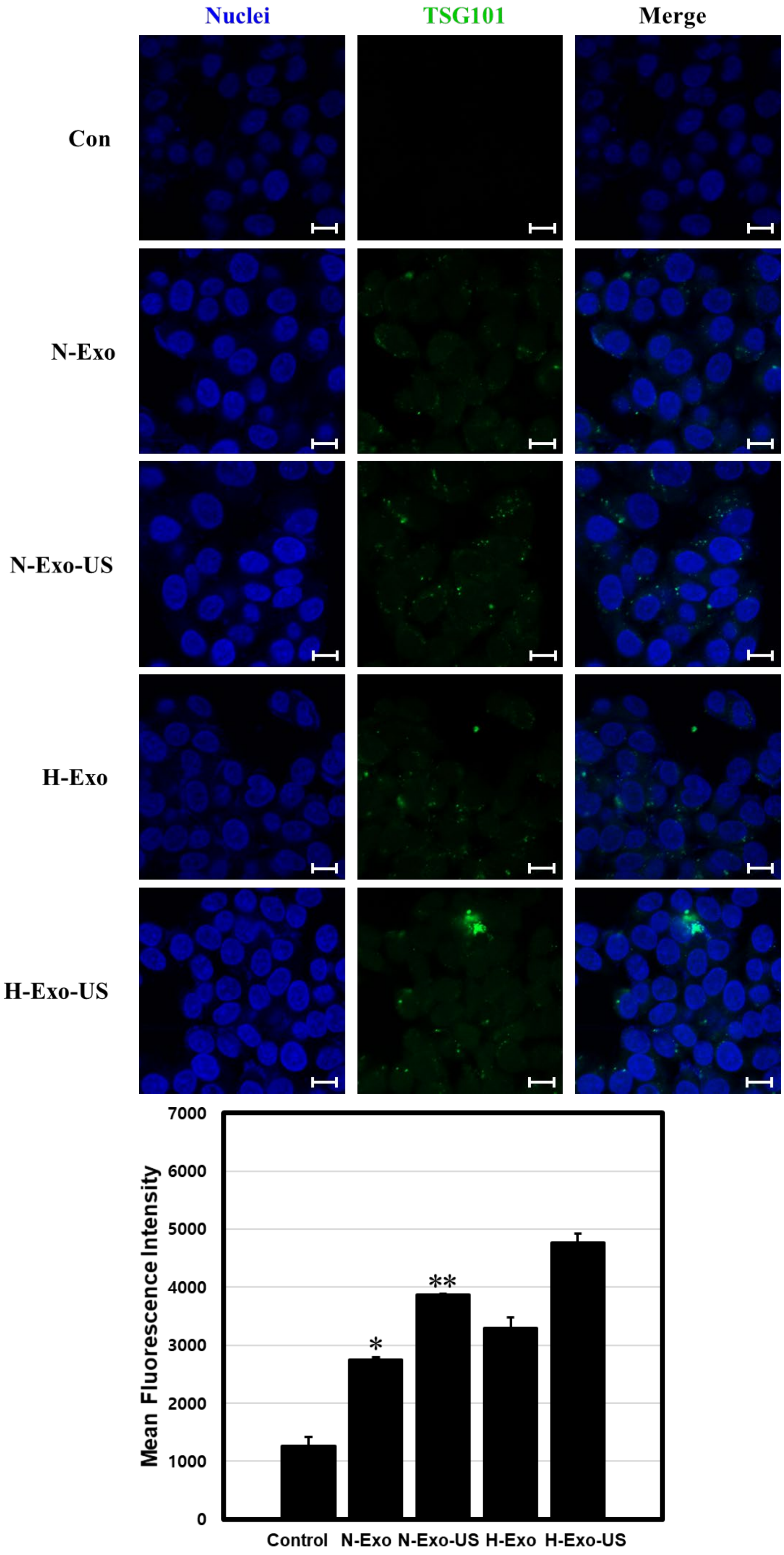

Uptake of dye-labeled exosomes into HepG2 cells monitored by confocal laser scanning microscopy. Cell nuclei were labeled with DAPI (blue) signals, and exosomal membranes were labeled with common exosome marker TSG101-FITC (green) signals. Successfully uptake induced by ultrasound was observed. Confocal microscopy image scale bar : 10 μm.
